# Contingent sounds change the mental representation of one’s finger length

**DOI:** 10.1038/s41598-017-05870-4

**Published:** 2017-07-18

**Authors:** Ana Tajadura-Jiménez, Maria Vakali, Merle T. Fairhurst, Alisa Mandrigin, Nadia Bianchi-Berthouze, Ophelia Deroy

**Affiliations:** 1 UCL Interaction Centre (UCLIC), University College London, University of London, London, UK; 2 Universidad Loyola Andalucía, Department of Psychology, Seville, Spain; 3 Universidad Loyola Andalucía, Human Neuroscience Lab, Seville, Spain; 4 Centre for the Study of the Senses, School of Advanced Study, University of London, London, UK; 5 Faculty of Philosophy, Ludwig Maximilian University, Munich, Germany; 6 Department of Philosophy, University of Warwick, Coventry, UK; 7 Munich Center for Neuroscience, Ludwig Maximilian University, Munich, Germany

## Abstract

Mental body-representations are highly plastic and can be modified after brief exposure to unexpected sensory feedback. While the role of vision, touch and proprioception in shaping body-representations has been highlighted by many studies, the auditory influences on mental body-representations remain poorly understood. Changes in body-representations by the manipulation of natural sounds produced when one’s body impacts on surfaces have recently been evidenced. But will these changes also occur with non-naturalistic sounds, which provide no information about the impact produced by or on the body? Drawing on the well-documented capacity of dynamic changes in pitch to elicit impressions of motion along the vertical plane and of changes in object size, we asked participants to pull on their right index fingertip with their left hand while they were presented with brief sounds of rising, falling or constant pitches, and in the absence of visual information of their hands. Results show an “auditory Pinocchio” effect, with participants feeling and estimating their finger to be longer after the rising pitch condition. These results provide the first evidence that sounds that are not indicative of veridical movement, such as non-naturalistic sounds, can induce a Pinocchio-like change in body-representation when arbitrarily paired with a bodily action.

## Introduction

The way we represent our body size, the location of the different body parts and other characteristics depends on external information provided by a wide range of sensory modalities(see^1,2^ for recent reviews). The so-called Pinocchio illusion^3^ is an often-cited example of how flexible the representation of the body can be in response to synchronous multisensory cues (e.g. refs^4,5^). In the classical version of this illusion, participants touch their nose while a muscle vibrator placed on participants’ bicep tendon induces the illusory feeling of one’s arm extending, thus altering the perceived position of one’s hand in space. This induced-conflict between synchronous tactile and proprioceptive cues leads to an elongation of one’s represented nose^5^. Could a similar illusion be generated by exteroceptive auditory cues that do not provide information about one’s body?

Though the role of vision, proprioception and touch in shaping body-representation has been the focus of past studies (e.g. refs^6,7^), the role of auditory input has only been recently explored. Even if we are not aware of it, certain sounds are nonetheless used to process position and location of one’s body in space, and can eventually lead to the updating of body-representations. Manipulating the quality, pitch, location and intensity of sounds that are produced when our hands or feet come into contact with a surface can alter the perceived material properties of the body^8,9^, the perceived length and position of limbs^10–12^, perceived body weight^13^ and other bodily sensations^14^. All of these effects rely on the fact that people extract spatial and material information from impact sounds that are produced in synchrony with their movements or when manipulating objects. But what happens with sounds that provide no information about the impact produced by or on the body? Will a non-naturalistic auditory change, lead to a change in body-representation, if it is paired with one’s body or a specific body part? Changes in pitch are not typically associated with bodily movements and yet are readily interpreted as a change in height or size(see^15^ for recent review). As such, they represent an ideal candidate to test whether non-naturalistic sounds will lead to a body illusion similar to those illusions resulting from the manipulation of the naturalistic sounds produced by familiar actions and actual movements.

Pitch may be defined as the most salient perceptual dimension corresponding to the physical fundamental frequency of a sound. Though changes in pitch are indicative of displacement of objects in the horizontal plane (‘the Doppler effect’) evidence shows that they are also readily interpreted as changes in the vertical plane^15^. Not only do we use a spatial terminology to refer to ‘high’/‘low’ pitch and ‘ascending’/‘descending’ tones, but we also map pitch onto spatial height^16^. Evidence shows that pitch of sounds can bias performance on a variety of spatial tasks, even though it does not provide spatially relevant information^17–20^. For instance, pitch influences the perceived sound location, with the sources of higher pitches being positioned higher on the vertical axis than those of lower pitches^21,22^. Further, changes or differences in pitch, although apparently uninformative about the direction or location of visual targets, can modulate performance on speeded detection tasks^20,23^ and visual search of targets at various locations^24^, or generate illusions of visual movements^25,26^. This crossmodal correspondence between pitch and spatial height develops at a very early age (i.e. in preverbal infants)^27^ and can be also found in the few populations that do not use spatial language to describe auditory pitch^16^, which suggests that language is not necessary for the association between space and pitch^15^. Recent evidence shows that a sound with rising pitch not only corresponds to an upward visual movement, but also to an upward tactile movement^28^ (see also the report of a static tone/upper tactile location correspondence^29^). Besides this correspondence with height, pitch is also associated to physical size in the visual domain, with static high and low pitches being respectively congruent with smaller and larger visual size^23,30–33^, and ascending and descending pitches being respectively congruent with growing and shrinking size^34^.

Based on this evidence, we hypothesized that pitch changes, although not previously associated with impact or body movement, could lead to body-representation changes if paired and presented synchronously with a bodily action. Here, we report two experiments in which we tested this hypothesis. In both experiments, we removed the visual influence by covering participants’ arms and hands with a black cloak, and we asked participants to pull on the tip of their right index finger with their left arm. Experiment 1 explored the effect of listening to sounds of rising, falling or constant pitch while pulling on one’s occluded fingertip on the represented finger size. We also evaluated the effects of the different sounds on the estimated vertical position of the finger (i.e., fingertip and knuckle positions), as changes in pitch may lead to the illusion of vertical displacement of one’s finger position independently of the effects on the represented finger size. Experiment 2 explored whether the effects observed in Experiment 1 are dependent on the direction of finger pulling. An additional control experiment was run to confirm that the different sounds employed in Experiments 1 and 2 do not differently affect the pulling strength the participants apply on their finger (see details in Supplementary material).

**Figure 1:**
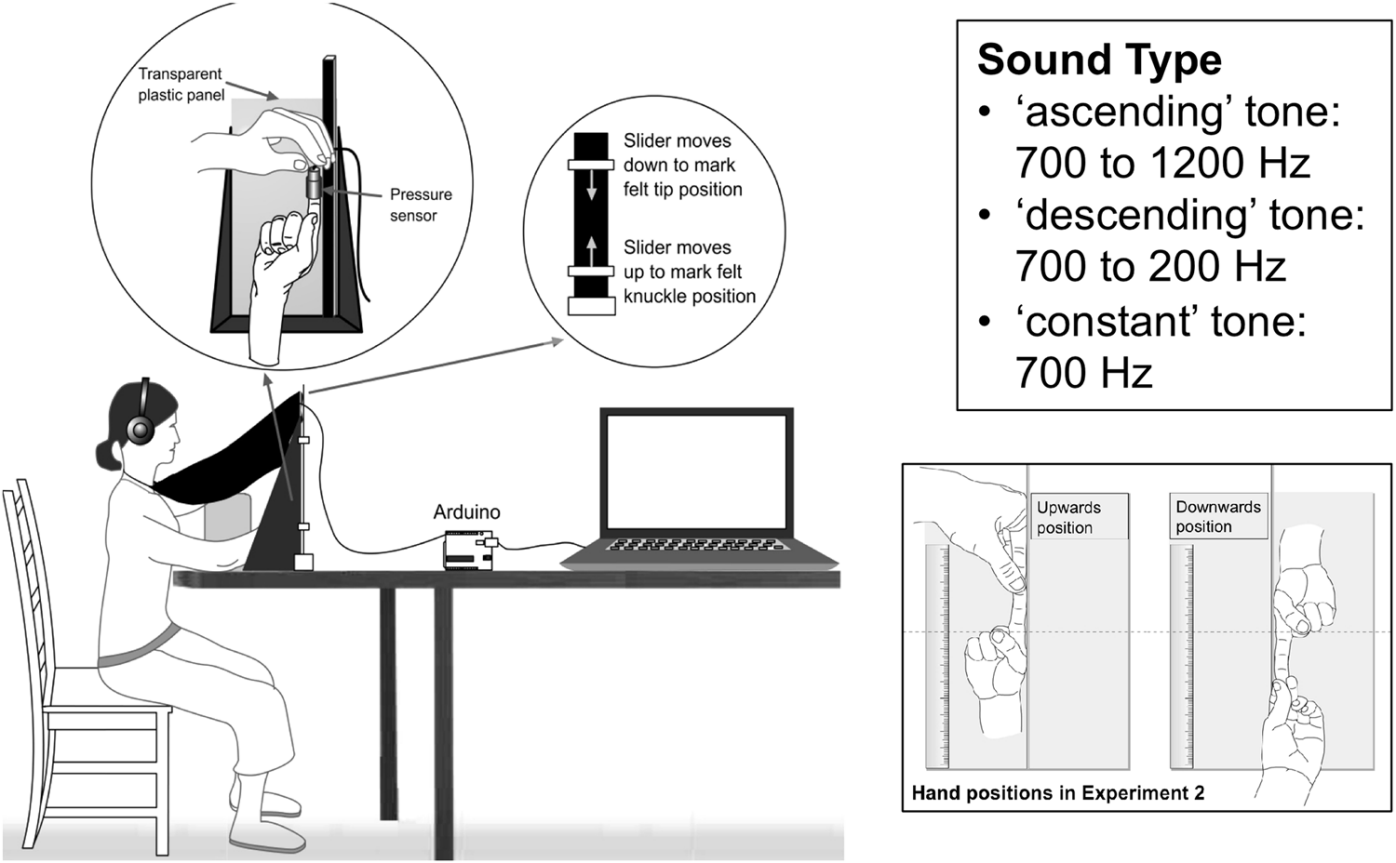
Experimental setup and design: A black cloak was used to hide the hand from the participant’s view. Participants were asked to hold their index finger still and straight. On each trial, the pulling of the finger was detected by a pressure sensor and simultaneously triggered a 2000-ms pure tone (‘ascending’, ‘descending’ or ‘constant’ in Experiment 1, ‘ascending’ or ‘descending’ in Experiment 2) that participants listened to through headphones. After the sound ended, the task was to estimate the position of the fingertip and knuckle using the apparatus shown in the right circle. In Experiment 1 participants kept their index fingertip pointing upwards during all the experiment, which included 10 repetitions of each sound type. In Experiment 2 the position of the hand was varied between two experimental blocks (each block with 10 repetitions of each sound type), so that participants either kept the fingertip pointing upwards or downwards, as shown in the figure (note that the ruler is displayed for illustration purposes, but that it was blacked out on the side facing the participant).

Prior to each experiment, we used a reference static tone for participants to anchor the new pairing between the action of pressing one’s immobile fingertip and the production of a sound. This “anchor” task was based on the extensive literature highlighting the relative nature of crossmodal correspondences (for an overview see ref.^35^) and here had two purposes: first, to give a standard tone to help the participants to identify the two auditory stimuli used in the experiment as rising and descending, having been given a static tone; and, second, to create a new association between the static tone and the fingertip in order to encourage an association between the experimental sounds and the finger. In the experiments, participants pulled on their occluded fingertip while listening to sounds whose pitch either increased, decreased or remained constant by comparison with the reference static tone (see experimental design in Fig. 1).

Participants were asked to pull on their right index finger with the left hand – rather than merely apply pressure – in order to minimise conflict between proprioceptive information and the sound they heard. To counteract any potential physical effect resulting from the pulling of the finger, participants were asked to keep their right hand and arm immobile. After each sound, participants provided estimates of fingertip and knuckle positions, from which estimated finger length was calculated. A questionnaire was administered to capture subjective feelings about their finger after each sound condition.

The overall prediction was that the synchrony between the changing pitch and the pulling action would be sufficient to induce an illusory change in one’s represented finger size. Given the background literature on pitch size-correspondence and updates of bodily representations, three more specific predictions were made:

1. the represented finger would become longer in the rising pitch condition, while a shortening of the finger would not necessarily occur in the descending pitch condition given that illusory bodily reductions are harder to induce than bodily extensions^36^;
2. the change in the represented finger length would come from the illusory movement of the pulled fingertip raising (or descending) in space induced by the sound;
3. the change in the represented finger length would come from the rising (or descending) sound being interpreted as an overall change in size of the pulled finger.

The three predictions are compatible, though each could hold independently of the other two.

## Results

### Experiment 1

#### Estimates of fingertip position, knuckle position and finger length

**Figure 2:**
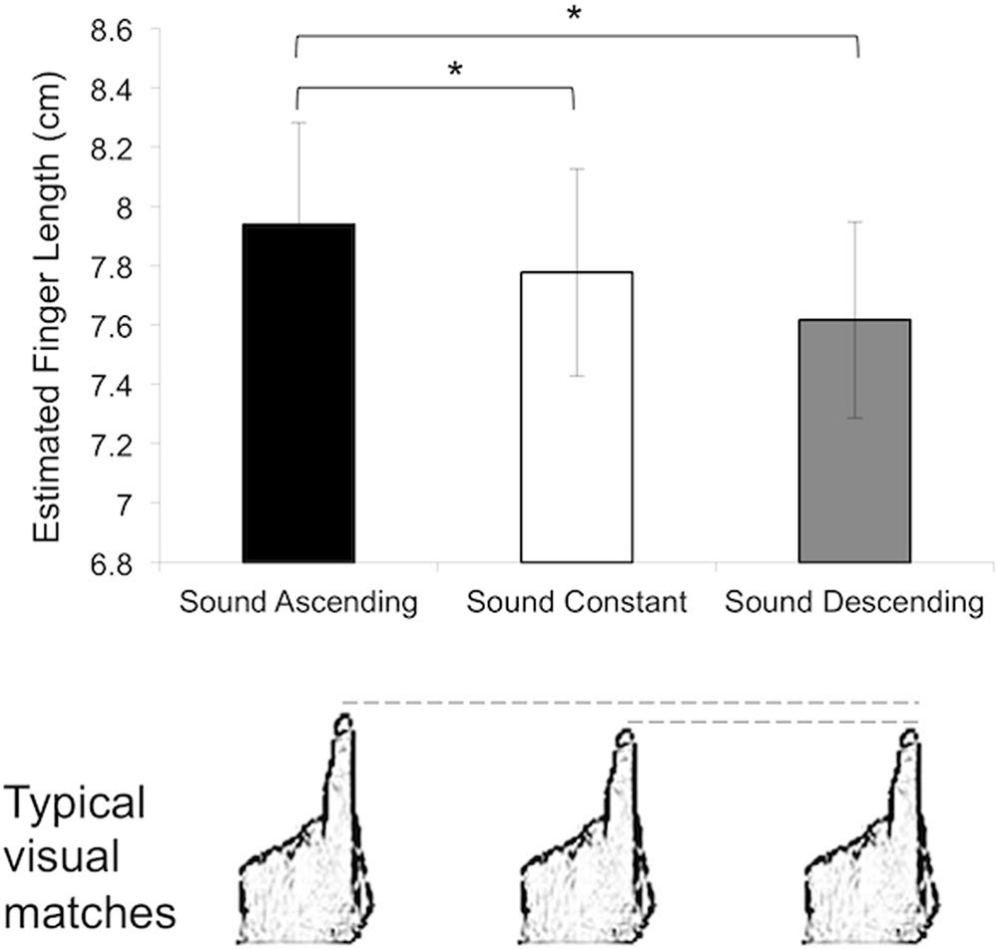
Results of Experiment 1: Mean (±s.e.m.) estimated finger lengths as a function of condition in Experiment 1 (N = 24). The asterisk indicates significant differences between sound conditions (*p* < 0.05, corrected for multiple comparisons). Participants provided larger finger length estimates for the ‘ascending’ sound, compared with the ‘descending’ sound and with the ‘constant’ sound. The bottom panel displays finger drawings chosen by participants to describe the subjective feeling of their finger when listening to the sound (the drawing closest to the mean choice is displayed as a function of condition). Participants selected significantly longer visual matches to their index finger after the ‘ascending’ sound as compared to the ‘constant’ and ‘descending’ sounds. Drawings in bottom panel are reprinted from Current Biology, 15(14), de Vignemont, F., Ehrsson, H. H. & Haggard, P., Bodily Illusions Modulate Tactile Perception, 1286-1290, Copyright (2005), with permission from Elsevier.

For each trial, the estimated knuckle position was subtracted from the estimated fingertip position in order to calculate the estimated finger length. For each estimate type (estimated finger length, fingertip position and knuckle position), data from trials exceeding three standard deviations from the mean group value were excluded. For those participants with only one trial per condition excluded (two participants), data for that trial was replaced by the mean value of the other nine trials for that condition. Those other (two) participants for whom more than one trial per condition had been eliminated were excluded from all analyses. Data analyses were performed then on the remaining data of 22 female participants (mean age = 20.05, range = 18–23). The mean estimates are displayed in Fig. 2.

Individual z-scores of these behavioural data were calculated in order to achieve normality (normality checked was performed with Shapiro-Wilk tests) and data from the 10 repetitions for each condition were averaged. These data on estimates of fingertip position, knuckle position and finger length were then submitted to three separate within-subjects analyses of variance (ANOVA) with ‘sound’ (‘ascending’, ‘descending’ or ‘constant’) as factor. These analyses showed a significant main effect of sound for finger length (*F*(2,42) = 4.80, *p* = 0.013, η^2^_p_ = 0.19); the effect size of the mean difference between conditions is large according to Cohen’s rule^37^. No effects of sound emerged for knuckle or fingertip position (see full summary of F-values and p-values in Table 1). Significant effects were further investigated with independent-samples t-tests, with the significance alpha level adjusted for multiple comparisons using Bonferroni correction. These t-tests revealed larger finger length estimates for the ‘ascending’ sound, compared with the ‘descending’ sound, *t*(21) = 2.77, *p* = 0.011, Cohen’s d = 1.07, and with the ‘constant’ sound, *t*(21) = 2.61, *p* = 0.016, Cohen’s d = 0.83. For both comparisons, the effect size of the difference between the two means is large according to Cohen’s rule^37^. No difference on estimated finger length was found between ‘descending’ and ‘constant’ sounds.

**Table 1:** Results from statistical tests on estimates of fingertip position, knuckle position and finger length in Experiment 1.

Dependent variable	ANOVA	Ascend vs. constant	Ascend vs. descend	Descend vs. constant
Estimated fingertip position	F(2,42) = 0.60 p = 0.554	—	—	—
Estimated knuckle position	F(2,42) = 1.30 p = 0.283	—	—	—
Estimated finger length	**F(2,42) = 4.80 p = 0.013**	**t(21) = 2.61 p = 0.016**	**t(21) = 2.77 p = 0.011**	t(21) = 0.96 p = 0.349

Results from ANOVAs comparing the effects of sound in the three conditions are in the second column. Significant effects were further investigated with independent-samples t-tests (with correction for multiple comparisons *α* = 0.017) and are displayed in columns three to five. Significant differences are marked in bold font.

#### Visual templates of index finger

An ANOVA on aligned rank transformed data, followed by Wilcoxon signed ranked tests, was used to explore the effect of ‘sound’. When asked to select a visual drawing to describe the subjective feeling of their finger when listening to the sound (see Fig. 2 – bottom panel), participants selected significantly larger visual matches to their index finger after the ‘ascending’ sound (median ratio was 8:7, range was 6:7 to 10:7) as compared to the ‘constant’ (*Z* = 3.62, *p* < 0.001; median ratio was 7:7, range was 4:7 to 8:7) and ‘descending’ (*Z* = 2.93, *p* = 0.003) sounds (*F*(2,63) = 9.11, *p* < 0.001; median ratio was 7:7, range was 3:7 to 10:7). No difference was found between ‘descending’ and ‘constant’ sounds.

#### Other subjective results

Questionnaire data were analysed with ANOVAs on aligned rank transformed data comparing the three sound conditions. Significant effects were further investigated with Wilcoxon tests, with the significance alpha level adjusted for multiple comparisons. For all statistical tests alpha level was set at 0.05, 2-tailed. Subjective reports (see full questionnaire data and analyses in Table 2) revealed that participants felt that their finger was longer to a larger extent in the ‘ascending’ sound conditions than in the ‘descending’ sound conditions (*Z* = 2.67, *p* = 0.008); they also felt that their finger was descending to a lesser extent (*Z* = 3.03, *p* = 0.002). Furthermore, in the ‘descending’ sound conditions participants felt that their finger was shorter to a larger extent than in the ‘constant’ sound conditions (*Z* = 2.63, *p* = 0.008). Note that results of correlational analyses between estimates and subjective reports are provided as supplementary material.

**Table 2:** Median (Range) for questionnaire data in Experiment 1.

While listening to the sound...	Ascend	Constant	Descend	ANOVA on aligned rank transformed data	Ascend vs. constant	Ascend vs. descend	Descend vs. constant
I felt pulling on my finger produced the sound	5.5 (1–7)	5 (1–7)	5 (1–7)	F(2,63) = 1.32 p = 0. 27	—	—	—
I felt my finger was longer	4 (1–7)	3 (1–6)	2.5 (1–6)	**F(2,63) = 3.87 p = 0.026**	Z = 2.06, p = 0.039	**Z = 2.67, p = 0.008**	Z = 1.44, p = 0.149
I felt my finger was shorter	2 (1–4)	2 (1–4)	4 (1–6)	**F(2,63) = 3.40 p = 0.039**	Z = .72, p = 0.470	Z = 2.32, p = 0.021	**Z = 2.63, p = 0.008**
I felt my finger was rising	4 (1–6)	2.5 (1–7)	3 (1–5)	F(2,63) = 1.62 p = 0.21	—	—	—
I felt my finger was descending	2 (1–4)	2 (1–6)	3.5 (1–6)	**F(2,63) = 3.63 p = 0.032**	Z = 1.98, p = 0.048	**Z = 3.03, p = 0.002**	Z = 2.09, p = 0.037
my finger felt stretched	5.5 (1–7)	3.5 (1–7)	4 (1–7)	F(2,63) = 3.11 p = 0.052	—	—	—
my finger felt squashed	2 (1–6)	2 (1–6)	3 (1–5)	F(2,63) = 0.85p = 0.43	—	—	—
I couldn’t tell how long my finger was	4 (1–6)	4 (2–6)	4 (1–6)	F(2,63) = 0.36 p = 0.70	—	—	—
I couldn’t locate the position of my knuckle	3 (1–6)	3 (1–6)	3 (1–6)	F(2,62) = 0.13 p = 0.87	—	—	—
I couldn’t locate the position of my fingertip	3 (1–6)	2.5 (1–6)	3 (1–6)	F(2,63) = 0.11 p = 0.89	—	—	—
the feeling from my finger was unexpected	3 (1–5)	2 (1–7)	2 (1–5)	F(2,63) = 0.05 p = 0.95	—	—	—
my finger felt like it wasn’t my own	1.5 (1–4)	2 (1–6)	2 (1–4)	F(2,63) = 0.24 p = 0.78	—	—	—
my finger felt numb	3 (1–7)	1.5 (1–7)	3 (1–5)	F(2,63) = 0.34 p = 0.72	—	—	—

Participants rated their level of agreement with the statements using a 7-item Likert scale (1 to 7). Results from ANOVA on aligned rank transformed data comparing three conditions are in the fifth column. Significant differences are marked in bold font. Significant tests were followed by Wilcoxon signed ranked tests that are presented in columns six-eight (with correction for multiple comparisons α = 0.017).

### Experiment 2

#### Estimates of fingertip position, knuckle position and finger length

**Figure 3:**
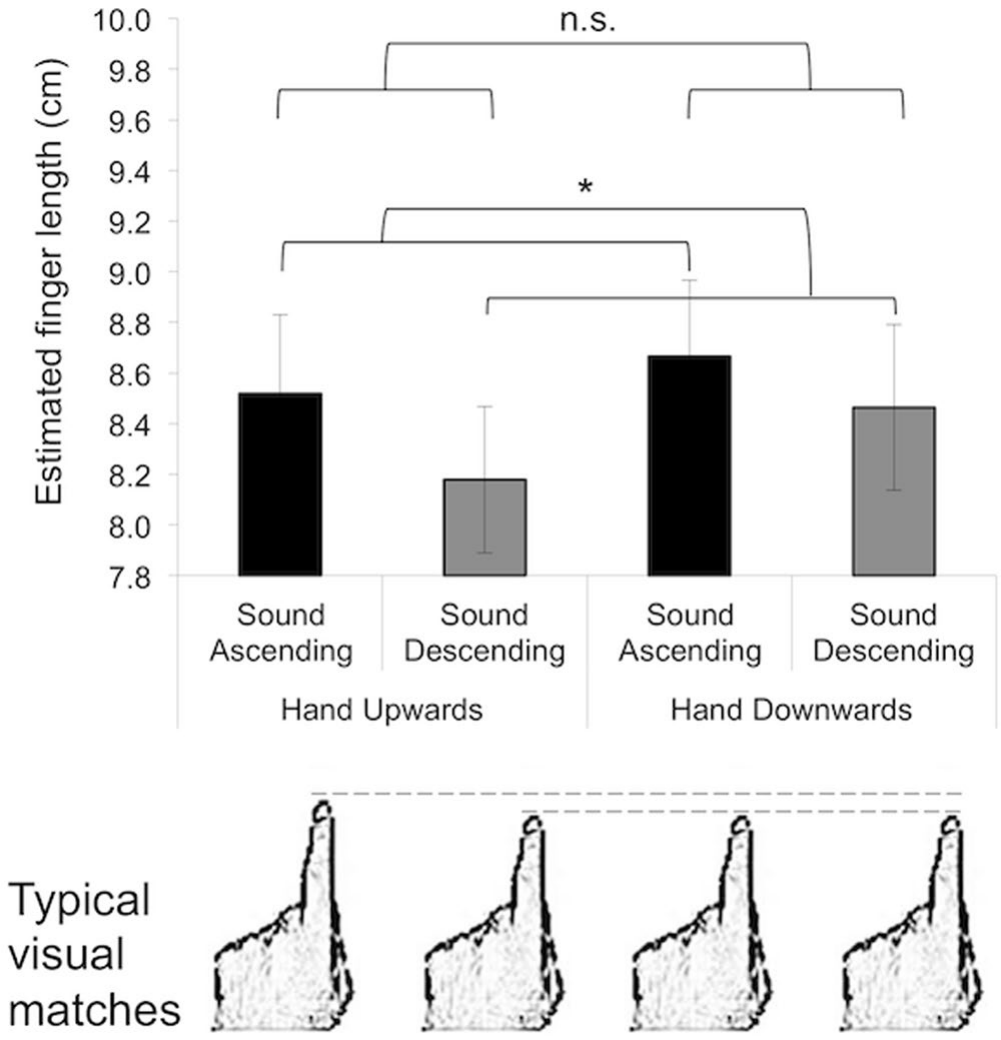
Results of Experiment 2: Mean (±s.e.m.) estimated finger lengths as a function of condition in Experiment 2 (N = 24). The asterisk indicates significant differences between sound conditions (*p* < 0.05, corrected for multiple comparisons).n.s. refers to non-significant differences between sound conditions. Longer finger length estimates were found for the ‘ascending’ sound, as compared with the ‘descending’ sound. No effects of pointed direction were found. The bottom panel displays finger drawings chosen by participants to describe the subjective feeling of their finger when listening to the sound (the drawing closest to the mean choice is displayed as a function of condition). Participants selected significantly larger visual matches to their index finger after the ‘ascending’ as compared to the ‘descending’ conditions and after the ‘upwards’ as compared to the ‘downwards’ finger position. No interaction effect was found between ‘finger position’ and ‘sound type’. Drawings in bottom panel are reprinted from Current Biology, 15(14), de Vignemont, F., Ehrsson, H. H. & Haggard, P., Bodily Illusions Modulate Tactile Perception, 1286-1290, Copyright (2005), with permission from Elsevier.

Similar data analyses to the ones in Experiment 1 were performed. For those participants with only one trial per condition excluded (six participants), data for that trial was replaced by the mean value of the other nine trials for that condition. Those other participants (two participants) with data missing from more than one trial per condition were excluded from all analyses. Data analyses were performed then on the data of 22 female participants (mean age = 21.7, range = 19–26). 10 had the ‘upwards’ position first, and 12 hand the ‘downwards’ position first. The mean estimates are displayed in Fig. 3.

Individual z-scores of these behavioural data were calculated in order to achieve normality (normality checked was performed with Shapiro-Wilk tests) and data from the 10 repetitions for each condition were averaged. These data on estimates of fingertip position, knuckle position and finger length were then submitted to three separate 2 × 2 ANOVAs with ‘finger position’ (‘upwards’ or ‘downwards’) and ‘sound’ (‘ascending’ or ‘descending’) as within-subjects factors and block order (‘upwards first’, ‘downwards first’) as a between-subjects factor. No effects of block order emerged. The analyses on the finger length data showed a significant main effect of sound (*F*(1,20) = 7.48, *p* = 0.013, η^2^_p_ = 0.272), revealing larger finger length estimates for the ‘ascending’ sound, compared with the ‘descending’ sound; the effect size of the mean difference between conditions is large according to Cohen’s rule^37^. Neither the effect of finger position or its interaction with sound reached significance (see full summary of F-values and p-values in Table 3). The analyses on fingertip position showed a significant main effect of finger position (*F*(1,20) = 506015.52, *p* < 0.001, η^2^_p_ = 1.00), revealing that fingertip position was estimated to be at a higher position in the ‘upwards’ than in the ‘downwards’ position; this effect was expected because the actual fingertip position indeed changed across ‘upwards’ and ‘downwards’ blocks while the actual knuckle position was kept fixed. No effects emerged for knuckle position and no significant two-way interactions between sound and finger position emerged for any of the variables.

**Table 3:** Results from statistical tests on estimates of fingertip position, knuckle position and finger length in Experiment 2.

Dependent variable	Effect of Finger Position (Upwards vs Downwards)	Effect of Sound (Ascending vs Descending	Interaction Finger Position * Sound
Estimated fingertip position	**F(1,20) = 506015.52 p < 0.001**	F(1,20) = 0.01 p = 0.929	F(1,20) = 1.13 p = 0.301
Estimated knuckle position	F(1,20) = 0.39 p = 0.539	F(1,20) = 0.23 p = 0.634	F(1,20) = 1.88 p = 0.186
Estimated finger length	F(1,20) = 0.49 p = 0.490	**F(1,20) = 7.48 p = 0.013**	F(1,20) = 0.87 p = 0.362

Results from ANOVAs comparing the effects of finger position and sound are, respectively, in the second and the third column, while the interaction effects between finger position and sound are in the fourth column. Significant differences are marked in bold font.

#### Visual templates of index finger

Participants’ responses were submitted to an ANOVA on aligned rank transformed data with ‘finger position’ and ‘sound’ as within-subjects factors. Participants selected significantly larger visual matches to their index finger after the ‘ascending’ as compared to the ‘descending’ conditions (*F*(1,80) = 5.80, *p* = 0.0018), and after the ‘upwards’ as compared to the ‘downwards’ position (*F*(1,80) = 5.25, *p* = 0.025; see Fig. 3 – bottom panel). No interaction effect was found between ‘finger position’ and ‘sound’. Median ratios and ranges for all conditions were: ‘upwards – ascending’: median ratio 8:7, range 5:7 to 11:7; ‘downwards – ascending’: median ratio 7:7, range 5:7 to 10:7; ‘downwards – ascending’: median ratio 7:7, range 4:7 to 10:7; ‘downwards - descending’: median ratio 7:7, range 4:7 to 10:7.

#### Other subjective results

Questionnaire data were analysed with ANOVAs on aligned rank transformed data. These subjective reports (see full questionnaire data and analyses in Table 4) confirmed again that, in the ‘ascending’ conditions participants felt that their finger was longer (*F*(1,84) = 11.44, *p* = 0.001), that it was rising (*F*(1,84) = 11.33, *p* = 0.001) and that it stretched (*F*(1,84) = 7.73, *p* = 0.007) to a larger extent than in the ‘descending’ conditions, and they felt that their finger was shorter (*F*(1,84) = 5.08, *p* = 0.027) and that it was descending to a lesser extent (*F*(1,84) = 11.60, *p* = 0.001). Further, in the ‘upwards’ conditions participants felt, to a larger extent than in the ‘downwards’ conditions, that their finger was rising (*F*(1,84) = 7.69, *p* = 0.007) and they felt to a lesser extent that their finger was descending (*F*(1,84) = 23.50, *p* < 0.001). Results of correlational analyses between measures are provided as supplementary material.

**Table 4:** Median (Range) for questionnaire data in Experiment 2.

While listening to the sound...	Upw –Ascend	Upw - Descend	Downw - Ascend	Downw - Descend	Effect of Finger Position (Upwards vs. Downwards)	Effect of Sound (Ascend vs. Descend)	Interaction Finger Position * Sound
I felt pulling on my finger produced the sound	5 (1–7)	5 (1–7)	5 (1–7)	4.5 (1–7)	F(1,84) = 0.04 p = 0.850	F(1,84) = 0.74 p = 0.850	F(1,84) = 0.07 p = 0.799
I felt my finger was longer	5 (1–7)	3 (1–6)	4 (1–6)	3 (1–6)	F(1,84) = 1.16 p = 0.285	**F(1,84) = 11.44 p = 0.001**	F(1,84) = 1.43 p = 0.236
I felt my finger was shorter	1 (1–4)	2.5 (1–6)	2.5 (1–5)	3 (1–6)	F(1,84) = 2.21, p = 0.141	**F(1,84) = 5.08 p = 0.027**	F(1,84) = 1.47 p = 0.229
I felt my finger was rising	5 (1–7)	2.5 (1–6)	3.5 (1–6)	2 (1–6)	F(1,84) = 7.69 p = 0.007	**F(1,84) = 11.33 p = 0.001**	F(1,84) = 0.80 p = 0.372
I felt my finger was descending	1 (1–3)	3 (1–6)	3.5 (1–6)	4 (1–6)	F(1,84) = 23.50 p < 0.001	**F(1,84) = 11.60 p = 0.001**	F(1,84) = 1.57 p = 0.213
my finger felt stretched	5 (1–7)	3 (1–7)	5 (1–6)	3 (1–7)	F(1,84) = 0.015 p = 0.902	**F(1,84) = 7.73 p = 0.007**	F(1,84) = 1.39 p = 0.242
my finger felt squashed	1 (1–6)	2 (1–6)	2 (1–6)	1.5 (1–6)	F(1,84) = 0.12 p = 0.733	F(1,84) = 0.70 p = 0.404	F(1,84) = 1.75 p = 0.189
I couldn’t tell how long my finger was	4 (1–7)	4 (2–7)	4 (2–7)	4 (1–7)	F(1,84) = 0.13 p = 0.723	F(1,84) = 0.55 p = 0.462	F(1,84) = 0.43 p = 0.514
I couldn’t locate the position of my knuckle	3.5 (1–7)	4 (1–7)	3 (1–7)	3 (1–7)	F(1,84) = 0.27 p = 0.605	F(1,84) = 0.48 p = 0.489	F(1,84) = 0.12 p = 0.730
I couldn’t locate the position of my fingertip	4 (2–7)	4 (1–7)	3 (1–7)	3 (1–7)	F(1,84) = 1.15 p = 0.286	F(1,84) = 0.10 p = 0.749	F(1,84) = 0.38 p = 0.537
the feeling from my finger was unexpected	2 (1–7)	2 (1–7)	2 (1–5)	2 (1–7)	F(1,84) = 0.55 p = 0.458	F(1,84) = 0.01 p = 0.908	F(1,84) = 0.73 p = 0.394
my finger felt like it wasn’t my own	2 (1–7)	2 (1–7)	2 (1–7)	2 (1–7)	F(1,84) = 0.19 p = 0.667	F(1,84) = 0.02 p = 0.886	F(1,84) = 0.19 p = 0.661
my finger felt numb	2 (1–6)	2 (1–6)	2 (1–6)	2 (1–5)	F(1,84) = 0.21 p = 0.649	F(1,84) = 0.28 p = 0.596	F(1,84) = 0.79 p = 0.376

Results from ANOVA on aligned rank transformed data looking at the effects of finger position, sound condition and their interaction are in columns sixth to eight. Significant effects are marked in bold font.

## Discussion

Results demonstrate an ‘auditory Pinocchio’ effect, with participants estimating the length of their finger to be longer after a two-second rising pitch sound, accompanied by pulling of their fingertip, as contrasted with either a descending pitch or with a constant tone. Crucially, though there were changes in the absolute estimated location of the fingertip, these do not explain the whole effect of sound, indicating an illusory finger extension independent of the illusory movement. Experiment 2 shows that the effect holds across hand positions, and upward or downward direction of finger pulling. This confirms that the contrast between the rising and descending sounds, and not the direction of the pulling movement exerted by the other hand, drives the illusion. Changes in body-representation were also evidenced in subjective reports. Participants selected drawings of a significantly longer finger as a match for their own after listening to the ‘ascending’ sound, as compared to the other sounds. The questionnaire corroborated these results.

Besides proposing an embodied analogue to the film adaptations of Pinocchio, where rising pitch sounds are used to accompany the extension of the character’s nose, the study also contrasts with the dominant reliance on vision in studying the plasticity of body-representations. Audition is a newcomer in this field, with recent studies documenting its role in the shaping and updating of one’s body-representations. While existing studies all draw on naturalistic sounds^8–13^, similar to those produced when one moves or interacts with physical objects, our results demonstrate for the first time that the effects of sounds on represented body size are not limited to such cases. Moreover, we demonstrate that new audio-tactile contingencies can affect represented body size in a relatively short amount of time. Other illusions introducing visuo-tactile conflict with the human hand require longer exposure to stimuli to achieve significant effects, such as in the case of the rubber-hand illusion, which arises approximately after 11 seconds of stimulation^38^ (note that most studies on the rubber-hand illusion employ periods of stimulation of at least 60 seconds, e.g. refs^6,7^). These visuo-tactile illusions are also partly constrained by the congruence between the visual stimuli and one’s previous experience of one’s own body^39^. By contrast the change in pitch used here is not formerly associated to one’s body or indicative of a veridical displacement of one’s body in space.

The explanation of the effect probably needs to appeal to the robust mappings that exist for dynamic pitch changes. As mentioned earlier, two correspondences, between dynamic pitch changes and vertical motion, and between change in pitch and change in size, affect behaviour automatically and universally^15,17,19,27,40^. The former mapping has been mostly demonstrated across audition and vision, but it also applies across audition and touch^28^. Recent findings suggest that the ear-filtering properties and sound localization processes are fine-tuned to mirror a frequency-elevation mapping found in natural auditory scenes and to change with proprioceptive and vestibular cues as head position changes^19^. When it comes to perceiving motion in the vertical plane, spectro-temporal cues play a bigger role than binaural cues, and the filtering properties of the pinna become relevant to detect differences in frequency^41^. Neuroimaging studies have revealed that rising/falling pitch operate at an intermediate level of the cortical hierarchy with respect to spatial words (such as ‘LEFT’ or ‘UP’) and to sounds actually moving: while moving sounds activate audio-visual motion areas (hMT+/V5+) and spatial words activate the right intraparietal sulcus (a higher-level convergence region) the effects of rising and descending pitch could be seen in both areas^42^. The activated superior parietal regions that are implicated in spatial processing^42^ overlap with multisensory parietal areas integrating somatosensory, visual and auditory signals to form body-representations^43^, which suggests an interaction between sound localization driven by changes in pitch and internal models of body-representation.

We show that a changing pitch led to a change in estimated finger length rather than a change in estimated vertical position, showing that body-representation of size is updated to conform to the proprioceptive information provided by the immobile forearm. These findings are distinct but compatible with the second mentioned correspondence between rising pitch and an overall increase in visual size^34^. Whether the present “auditory Pinocchio” effect is driven by touch and/or proprioception is open to further testing. In our experiments, we asked participants to pull their right index finger, rather than merely apply pressure to it with the left hand, to minimise any multisensory conflict that might cancel out the Pinocchio effect. In the literature, dynamic sound stimuli (rising/descending pitch) have been shown to be effective at inducing crossmodal effects when paired with dynamic visual stimuli^27,34,44,45^. A dynamic pulling action was therefore chosen here as the closest analogue. A further open question, therefore, is whether the pulling action is necessary to induce the effect, or whether mere pressure on or contact with the fingertip paired with a synchronous increasing pitch would be sufficient. A question also opened for further testing is the stronger effects for the rising than for the falling pitch that our study revealed, and which other studies on spatial crossmodal cueing of pitch on visual targets have similarly found^46,47^. Apart from this reported asymmetry on the crossmodal cueing of pitch, in the literature on sensory-driven changes in represented body size there are many fewer reports of illusory body shrinkage than of body expansion^36^ (although see refs^48,49^). Some authors have suggested that this asymmetry may reflect the fact that people are more used to experience the enlargement, as opposed to the shrinkage, of their body parts, for instance, during normal growth^36^. One further open question is whether the reported effects would be stronger if tactile and auditory stimuli would be spatially coincident, which could be achieved by using spatialization techniques over headphones or by using a loudspeaker next to the finger. We expect that the effects we found would hold when using loudspeakers as previous studies looking at the mapping between sound pitch and spatial elevation have used either headphones^25,42^ or loudspeakers^22^. The present method produced sizable effects^37^ and identified significantly larger finger length estimates for only one of the conditions - the ascending sound condition. Further, the effect held across two experiments, with two different experimenters (who were blind to the experimental conditions) and two different sets of participants, providing support to the robustness of the effect. However, future studies may consider using some sort of digital marker or using video coding to avoid the experimenter lag and increase sensitivity.

All these effects could be tested with various populations on other body parts, where sound might generate changes in length, width or both. The “auditory Pinocchio” effect reveals the strength of the auditory modulation of body-representation, and opens new possibilities to use arbitrary sounds in therapeutic or virtual settings. Sound feedback is increasingly seen as promising in such settings, especially when an individual is on the move or when looking at body parts may be detrimental to the rehabilitation process. This is the case, for instance, of rehabilitation of poor proprioception during gait or other movements in the elderly, in people with Parkinson’s Disease, in people who suffered a stroke or in people with chronic pain, who sometimes are reluctant to look at their affected body part^50–55^. Our study here adds to this by suggesting that auditory-driven strategies do not need to rely on visual or muscle stimulation, nor be constrained to actions that naturally produce sound (e.g., footsteps).

## Methods

### Experiment 1

#### Participants

Twenty-four female participants took part (mean age ± s.d.: 20.0 ± 1.4 years; age range: 18–23 years. Note that only female participants were tested as in refs^7,14^). In both experiments described here all participants reported having normal hearing and touch, with no neurological disorders. They were naïve as to the purposes of the study. Participants were paid for their time and gave their informed consent prior to their inclusion in the studies. Both experiments were conducted in accordance with the ethical standards laid down in the 1964 Declaration of Helsinki and approved by the ethics committee of University College London.

#### Apparatus and stimuli

Three experimental auditory stimuli were created on Audacity software and consisted of pure tones (2000-ms duration and 44.1-kHz sample rate) of increasing (‘ascending’ tone: 700 to 1200 Hz), decreasing (‘descending’ tone: 700 to 200 Hz) or constant (‘constant’ tone: 700 Hz) frequency. We adopted the frequency ranges of the stimuli used by Deroy and colleagues^28^ to study audio-tactile correspondences between auditory changes in pitch and tactile direction of movement. It should be noted that while the chosen frequency ranges differ in their starting/ending frequencies and their average frequency, these parameters are less significant than the direction of the frequency change in terms of mapping with changes in spatial elevation, as shown by Mossbridge and colleagues in a previous spatial cueing study^56^. Mossbridge and colleagues^56^ tested various frequency ranges differing in the initial, average and final frequency and found that these parameters had no effect on the robust cross-modal effects of ascending and descending frequency changes on guiding spatial attention. An additional auditory stimulus consisting of a pure tone (‘anchoring tone’: 250-ms duration, 700 Hz) was used in the “anchor” session that participants performed prior to the experimental session, as explained below. A 10-ms onset/offset ramp was applied to the auditory stimuli to prevent clipping. For all auditory stimuli sound level was set at 60 dBA.

A force-sensitive resistor (FSR; 4 mm-diameter active sensing area) attached to the participant’s right index fingertip was used to detect the participant’s finger pulling action and trigger the auditory stimulation. The FSR was connected to an Arduino Uno microcontroller linked to a computer. Presentation® software was used to control the stimulus delivery and to record the participant’s responses.

The experimental setup is illustrated in Fig. 1. This setup consisted of a transparent plastic panel (30 × 42 cm) attached on the bottom and right side to a metal stand that has a 2 × 2 × 50 cm vertical metal bar. Participants were seated in a chair and wore a pair of closed headphones with high passive ambient noise attenuation (Sennheiser HDA 300). They were asked to hold their index finger still and straight, fingertip pointing upwards, with the dorsum of the finger pressed against the transparent panel and the right side of the finger pressed against the metal bar. Their right arm rested in a cardboard ramp placed under the hand. Their left elbow rested on foam cushions to avoid fatigue during the experimental session in which they used the left hand to pull their right index finger. A black cloak was used to hide the hand from the participant’s view. This cloak was attached to the participant’s neck and covered the plastic frame, ramp and hand. A coloured clip was fixed on the top centre of the plastic panel (over the cloak) and served as the centre fixation point for participants.

A specially designed apparatus was used to collect participants’ estimates of their fingertip and knuckle position. This apparatus consisted of a 50-cm ruler fixed on the right side of the metal bar, parallel to it and 10 cm away. The ruler was blacked out on the side facing the participant. Two horizontal clips were mounted on the ruler, each of them with a red dot which served as visual points that the participant used to mark their felt fingertip and knuckle positions, as explained below. The two horizontal clips on the ruler were initially positioned at the heights of 10 cm and 50 cm. The height of participants’ index finger knuckle with respect to this ruler was approximately 25 cm, given that the cardboard ramp kept the hand elevated.

#### Experimental procedure

Verbal and written instructions about the tasks were given to participants at the beginning of the session. First, participants were asked to complete the “anchor” task, which consisted of pressing their right index fingertip with their left hand twenty times, an action that triggered the ‘anchoring’ tone on each occasion. Note that during this task, participants were not exposed to the ‘ascending’ or ‘descending’ tones. Having completed the “anchor” task, participants were asked to complete the experimental block.

In the experimental block, participants were required to look straight at the fixation point and to perform the simple action of pulling their right index fingertip using their left hand, while keeping the right index finger in a fixed position (straight and pressed against the plastic panel and metal bar). The pulling action triggered one of the three experimental tones (‘ascending’, ‘descending’ or ‘constant’). Participants were asked to keep pulling their finger until the sound they heard was over. They could then relax their left hand, but were asked to keep holding their right finger with the left hand. They were then asked to estimate the position they felt their right fingertip and knuckle to be, by having the experimenter adjust the two visual points placed on the ruler clips. This was done by the experimenter moving the top clip downwards, at a constant speed, until the participants indicated with a “stop” signal that the upper visual point had reached the fingertip position. The experimenter then moved the bottom clip upwards, at a constant speed, until the participants indicated with a “stop” signal that the lower visual point had reached the knuckle position^57,58^ for similar procedures. The experimenter recorded the fingertip and knuckle positions using the measurements on the back of the ruler, rounding to the nearest 0.5 cm (e.g. ref.^59^). The clips were repositioned after each trial. After one practice trial (with ‘constant’ tone), the participant was asked to repeat the task for thirty subsequent trials. Each experimental tone (‘ascending’, ‘descending’, ‘constant’) was presented ten times. The order of trials was randomized across participants. After fifteen trials, participants were given the option to have a short break before continuing with the task.

After the experimental block was completed, participants were asked to repeat the task of pulling their finger while listening to a tone for three more trials, one trial for each sound condition, with the presentation order randomized across participants. Participants completed a questionnaire after each trial. This questionnaire assessed their subjective experience during the finger-pulling task. The questionnaire contained thirteen statements, adopted from our previous studies^10,13^. The list of statements is presented in Table 2. Participants rated their level of agreement with the statements using a 7-item Likert scale, ranging from 1 (strongly disagree) to 7 (strongly agree), with 4 referring to “neither agree, nor disagree”. In addition to these statements, participants were presented with a range of 9 figures representing finger length adapted from ref.^4^. Each figure showed a prototypical whole hand with the index finger selectively shrunk or elongated. Participants were asked to choose one of the figures to describe the subjective feeling of their finger when listening to the sound. The ratio of finger length was varied from 3:7 to 11:7 of the width of the pictured hand.

### Experiment 2

#### Participants

Twenty-four female participants took part (mean age ± s.d.: 21.7 ± 2.2 years; age range: 19–26 years).

#### Apparatus, stimuli and experimental procedure

The experimental auditory stimuli, apparatus and experimental procedure were identical to those in Experiment 1, with the following exceptions. First, in this case only the ‘ascending’ and ‘descending’ tones were used. Second, in this case the ruler was placed on the left instead of the right side of the plastic panel. Third, a 1 cm-thick metal bar was positioned 10 cm to the right of the ruler and attached to the surface of the plastic panel on the side facing the participant. Finally, participants did not rest their right arm in a cardboard ramp placed under their hand, but instead kept the hand elevated during the full experimental block.

Depending on the experimental block, participants either kept the fingertip pointing upwards or downwards (see Fig. 1). In the ‘upwards’ block, participants were asked to place their hand on the left side of the metal bar and to keep their index finger held straight, fingertip pointing upwards, with the dorsum of the finger pressed against the transparent panel and the right side of the finger pressed against the metal bar. In the ‘downwards’ block, participants were asked to place their hand on the right side of the metal bar and to keep their index finger held straight, fingertip pointing downwards, with the dorsum of the finger pressed against the transparent panel and the left side of the finger pressed against the metal bar. The height of participants’ index finger knuckle was fixed to be the same in the upwards and downwards blocks (height with respect to the ruler was approximately 25 cm).

As in Experiment 1, participants were first asked to complete the “anchor” task, in which they pressed their right index with the fingers of their left hand twenty times, an action that triggered the ‘anchoring’ tone. Next, participants were asked to complete two experimental blocks, one with the ‘upwards’ finger position and the other with the ‘downwards’ finger position. In each block each experimental tone (‘ascending’ or ‘descending’) was presented ten times, resulting in twenty trials per block. The order of trials was randomized across participants. Whether the ‘upwards’ block or the ‘downwards’ block was presented first was counterbalanced between participants. Participants were given the option to have a short break between blocks.

After both experimental blocks were completed, participants were asked to repeat the task of pulling their finger while listening to a tone for four more trials, one trial for each sound condition and for each finger position, with the presentation order randomized across participants. After each trial participants completed the same questionnaire employed in Experiment 1.

### Data Availability

The datasets generated during and analysed during the current study are available in the UK Data Service ReShare repository, http://reshare.ukdataservice.ac.uk/852739/.

## Electronic supplementary material

Supplementary material
