# Author Correction: Contingent sounds change the mental representation of one’s finger length

**DOI:** 10.1038/s41598-018-23229-1

**Published:** 2018-03-15

**Authors:** Ana Tajadura-Jiménez, Maria Vakali, Merle T. Fairhurst, Alisa Mandrigin, Nadia Bianchi-Berthouze, Ophelia Deroy

**Affiliations:** 1 UCL Interaction Centre (UCLIC), University College London, University of London, London, UK; 2 Universidad Loyola Andalucía, Department of Psychology, Seville, Spain; 3 Universidad Loyola Andalucía, Human Neuroscience Lab, Seville, Spain; 4 Centre for the Study of the Senses, School of Advanced Study, University of London, London, UK; 5 Faculty of Philosophy, Ludwig Maximilian University, Munich, Germany; 6 Department of Philosophy, University of Warwick, Coventry, UK; 7 Munich Center for Neuroscience, Ludwig Maximilian University, Munich, Germany

Correction to: *Scientific Reports* 10.1038/s41598-017-05870-4, published online 18 July 2017

The Acknowledgements section in this Article is incomplete.

“AT is supported by the ESRC grant ES/K001477/1 (“The hearing body”) and by a Spanish “Ministerio de Economía y Competitividad” Ramón y Cajal research contract (RYC-2014-15421). OD, MF and AM are supported by the AHRC RTS (“Rethinking the senses”) grant AH/L007053/1. We thank Nick Wilkinson for helping with data collection, Temitayo Olugbade for helping with EMG recordings, and Alejandro Galvao Carmona, Vincent Hayward, Milagrosa Sánchez Martín and Matthew Longo for their helpful suggestions.”

should read:

“AT was supported by the ESRC grant ES/K001477/1 (“The hearing body”) and by RYC-2014–15421 and PSI2016-79004-R (“MAGIC SHOES: Changing sedentary lifestyles by altering mental body-representation using sensory feedback”; AEI/FEDER, UE), Ministerio de Economía, Industria y Competitividad of Spain. OD, MF and AM were supported by the AHRC RTS (“Rethinking the senses”) grant AH/L007053/1. We thank Nick Wilkinson for helping with data collection, Temitayo Olugbade for helping with EMG recordings, and Alejandro Galvao Carmona, Vincent Hayward, Milagrosa Sánchez Martín and Matthew Longo for their helpful suggestions.”

