# Supplementary material for: Contingent sounds change the mental representation of one’s finger length
Source: Sci Rep. 2017 Jul 18;7:5748. doi: 10.1038/s41598-017-05870-4 (PMC5515978; doi:10.1038/s41598-017-05870-4)
Supplement: Supplementary file 1 — Supplementary material [file 41598_2017_5870_MOESM1_ESM.doc]

**Supplementary material of the paper: Contingent sounds change the mental representation of one’s finger length**

By A. Tajadura-Jiménez, M. Vakali, M.T. Fairhurst, A. Mandrigin, N. Bianchi-Berthouze & O. Deroy

**Control experiment: Effects of sound on the strength of the pulling action**

**METHODS**

***Participants***

Twenty-seven participants took part (mean age ± s.d.: 25.2 ± 4.8 years; age range: 18-36 years, 15 female and 12 male). All participants reported having normal hearing and touch, with no neurological disorders. They were naïve as to the purposes of the study. Participants were paid for their time and gave their informed consent prior to their inclusion in the studies. The experiment was conducted in accordance with the ethical standards laid down in the 1964 Declaration of Helsinki and approved by the ethics committee of University College London.

***Apparatus and stimuli***

The experimental auditory stimuli and apparatus were identical to those in Experiment 1, with the exception that in this experiment two pairs of 30x24mm surface electromyographic (EMG) sensors were attached to the first dorsal interosseous (FDI) and abductor pollicis brevis (APB) muscles of the participants’ left hand. These two muscles activate during a pinch task ^S1^. FDI muscle was chosen based on previous work which showed that for simple griping actions with a pinch grasp posture, as were employed in this study, the EMG signal at the FDI muscle correlates with the grip force ^S2^. Note that while both FDI and APB muscle activities were recorded, our analyses finally focused only on the EMG data from the FDI muscles ^as in S2^, because the signal at the APB muscle turned out unstable and the sensors occasionally fell down due to the griping action. The sensors connected wirelessly to a dedicated unit (BTS FREEEMG), which acquired the EMG data at a sample frequency of 1 kHz. Time stamps were recorded for synchronization with the stimulus delivery. Figure S1 shows an example of the EMG data recorded for one participant for the 30 trials composing the experimental block.


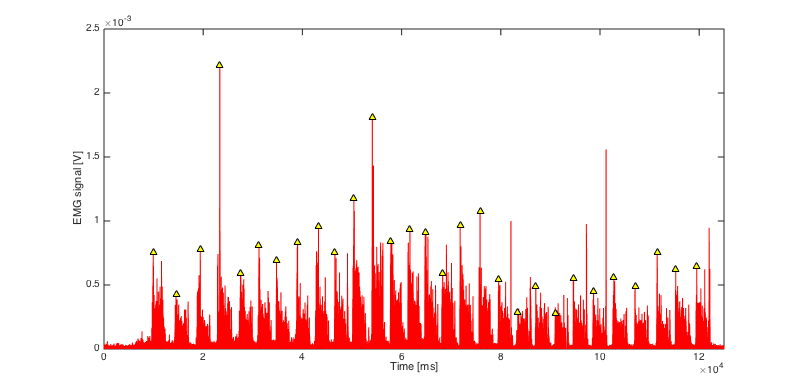


**Figure S1. EMG signal at the FDI muscle.** The displayed signal corresponds to the EMG data recorded for one participant for the 30 trials composing the experimental block. The yellow triangles mark the moment at which the sound stimuli were triggered.

***Experimental procedure***

The experimental procedure was similar to that in Experiment 1, with the exception that in the present experimental block participants were not asked to estimate the position they felt their right fingertip and knuckle to be. Instead, participants were fitted with EMG sensors to record the activity of the FDI and APG muscles of their left hand, as described above. Once these sensors were attached, participants were first asked to complete the “anchor” task. This was followed by the experimental block, in which they performed the action of pulling their right index fingertip using their left hand for thirty subsequent trials as in Experiment 1. The pulling action triggered one of the three experimental tones (‘ascending’, ‘descending’ or ‘constant’).

After the experimental block was completed, participants were asked to repeat the task of pulling their finger while listening to a tone for three more trials, one trial for each sound condition, with the presentation order randomized across participants. After each of these three trials participants rated their level of agreement with the statement *“I felt that the sound had an effect on the strength of my pulling action”* using a 7-item Likert scale, ranging from 1 (strongly disagree) to 7 (strongly agree), with 4 referring to “neither agree, nor disagree”. In addition to this statement, participants were presented with the same 9 visual templates representing finger length used in Experiment 1, and they were asked to choose one of the figures to describe the subjective feeling of their finger when listening to the sound.

Note that while both FDI and APB muscle activities were recorded, our analyses focused only on the EMG data on the FDI muscle, which follows the work by Wiertlewski and colleagues investigating the correlation between EMG signal and grip force ^2^.

**RESULTS**

***EMG results***

The FDI EMG data were rectified and then a smoothing filter was applied (Savitzky-Golay, window size 200, order 6) ^S2-S4^. For each participant, we calculated mean and peak scores of the EMG signal during the 2-second stimuli presentation for each trial. Data from the 10 repetitions for each condition were averaged.

Mean and peak EMG scores were submitted to two separate within-subjects analyses of variance (ANOVA) with ‘sound’ (‘ascending’, ‘descending’ or ‘constant’) as factor. No effects of sound emerged for mean EMG scores (*F*(2,52) = 0.399, *p* = 0.673; mean values ± SE were: 97.49 ± 20.46 µV (‘ascending’), 95.71 ± 21.85 µV (‘descending’) and 92.57 ± 19.59 µV (‘constant’)). Similarly, no effects of sound emerged for peak EMG scores (*F*(2,52) = 0.499, *p* = 0.610; mean values (± SE) were: 261.92 ± 76.49 µV (‘ascending’), 247.56 ± 80.36 µV (‘descending’) and 251.66 ± 72.95 µV (‘constant’)). These results suggest that the type of sound (ascending, descending or constant) does not affect the pulling strength the participants apply on their finger

***Subjective results***

Questionnaire data were analysed with two separate non-parametric Friedman tests with ‘sound’ as within-subjects factor. The analysis on the responses to the statement *“I felt that the sound had an effect on the strength of my pulling action”* did not reveal significant differences between sound conditions (*Chi^2^*(2) = 3.75, *p* = 0.154; mean values (± SE) were: 4.52 ± 0.28 (‘ascending’), 3.96 ± 0.28 (‘descending’) and 4.11 ± 0.26 (‘constant’)). By contrast, the analysis on the choices of visual templates of index finger revealed significant differences between sound conditions (*Chi^2^*(2) = 8.62, *p* = 0.013; mean values (± SE) were: 5.78 ± 0.29 (‘ascending’), 4.96 ± 0.27 (‘descending’) and 5.63 ± 0.27 (‘constant’)). This significant effect was further investigated with Wilcoxon one-tailed tests, with the significance alpha level adjusted for multiple comparisons (*p* = 0.033). These analyses showed that participants felt that their finger was longer to a larger extent in the ‘ascending’ sound conditions than in the ‘descending’ sound condition (*Z* = 2.16, *p* = 0.031). No significant differences were found between the other conditions.

**Table S1. Results from Spearman correlations between estimates of finger length and subjective reports in Experiments 1 and Experiments 2.** For each correlation test, Spearman’s Rho (rS) and p-value of the test are indicated. Significant correlations are displayed in bold and with gray shading.

| **Effect on subjective**  **reports** | **Experiment 1: effect on estimated finger length** | | | **Experiment 2: effect on estimated finger length** | | | |
| --- | --- | --- | --- | --- | --- | --- | --- |
| *While listening to the*  *sound …* | *Ascend* | *Constant* | *Descend* | *Upw –Ascend* | *Upw - Descend* | *Downw - Ascend* | *Downw - Descend* |
| I felt pulling on my finger produced the sound | **rS=-0.433** | rS=-0.155 | rS=0.361 | rS=-0.179 | rS=-0.349 | rS=0.369 | rS=0.072 |
|  | **p=0.044** | p=0.491 | p=0.098 | p=0.427 | p=0.112 | p=0.091 | p=0.751 |
| I felt my finger was longer | **rS=0.469** | rS=-0.017 | rS=0.076 | **rS=0.441** | rS=-0.160 | rS=0.093 | rS=-0.355 |
|  | **p=0.028** | p=0.939 | p=0.737 | **p=0.040** | p=0.478 | p=0.681 | p=0.105 |
| I felt my finger was shorter | rS=-0.064 | rS=0.191 | **rS=-0.615** | rS=-0.160 | rS=-0.122 | rS=0.234 | rS=-0.117 |
|  | p=0.777 | p=0.396 | **p=0.002** | p=0.477 | p=0.589 | p=0.294 | p=0.605 |
| I felt my finger was rising | rS=0.421 | rS=0.010 | rS=-0.136 | **rS=0.492** | rS=-0.066 | rS=0.276 | rS=0.014 |
|  | p=0.051 | p=0.964 | p=0.546 | **p=0.020** | p=0.771 | p=0.214 | p=0.950 |
| I felt my finger was  descending | **rS=0.557** | rS=0.164 | **rS=-0.502** | rS=-0.213 | rS=-0.036 | rS=-0.160 | rS=-0.303 |
|  | **p=0.007** | p=0.466 | **p=0.017** | p=0.341 | p=0.875 | p=0.478 | p=0.170 |
| my finger felt stretched | rS=0.052 | rS=-0.034 | rS=0.283 | rS=0.337 | rS=-0.077 | rS=0.094 | rS=-0.421 |
|  | p=0.817 | p=0.882 | p=0.202 | p=0.125 | p=0.732 | p=0.677 | p=0.051 |
| my finger felt squashed | rS=0.329 | rS=0.095 | **rS=-0.670** | rS=-0.194 | rS=-0.170 | rS=0.299 | rS=0.079 |
|  | p=0.135 | p=0.675 | **p=0.001** | p=0.388 | p=0.450 | p=0.176 | p=0.727 |
| I couldn't tell how long my  finger was | **rS=0.479** | rS=0.015 | **rS=-0.620** | rS=0.066 | rS=0.074 | rS=0.057 | rS=-0.138 |
|  | **p=0.024** | p=0.947 | **p=0.002** | p=0.770 | p=0.742 | p=0.802 | p=0.540 |
| I couldn't locate the position  of my knuckle | rS=0.184 | rS=0.106 | rS=-0.223 | rS=-0.238 | rS=0.065 | rS=0.075 | rS=0.193 |
|  | p=0.426 | p=0.637 | p=0.318 | p=0.287 | p=0.775 | p=0.741 | p=0.389 |
| I couldn't locate the position  of my fingertip | **rS=0.584** | rS=-0.068 | **rS=-0.664** | rS=-0.068 | rS=0.009 | rS=0.169 | rS=0.214 |
|  | **p=0.004** | p=0.765 | **p=0.001** | p=0.763 | p=0.968 | p=0.453 | p=0.340 |
| the feeling from my finger  was unexpected | rS=0.190 | rS=0.083 | **rS=-0.514** | rS=-0.061 | rS=-0.198 | rS=-0.197 | rS=0.041 |
|  | p=0.398 | p=0.712 | **p=0.014** | p=0.786 | p=0.378 | p=0.380 | p=0.855 |
| my finger felt like it wasn't  my own | rS=0.381 | rS=-0.322 | **rS=-0.678** | rS=0.034 | rS=-0.006 | rS=0.070 | rS=-0.083 |
|  | p=0.080 | p=0.143 | **p=0.001** | p=0.881 | p=0.977 | p=0.756 | p=0.715 |
| my finger felt numb | rS=0.388 | rS=0.241 | **rS=-0.489** | rS=0.055 | rS=-0.257 | rS=-0.017 | rS=-0.105 |
|  | p=0.075 | p=0.279 | **p=0.021** | p=0.806 | p=0.249 | p=0.940 | p=0.642 |
| Effect on finger drawings | rS=0.098 | rS=-0.195 | **rS=0.507** | rS=0.409 | rS=-0.095 | rS=0.268 | rS=-0.138 |
|  | p=0.665 | p=0.384 | **p=0.016** | p=0.059 | p=0.675 | p=0.240 | p=0.541 |

**Table S2. Results from Spearman correlations between estimates of fingertip position and subjective reports in Experiments 1 and Experiments 2.** For each correlation test, Spearman’s Rho (rS) and p-value of the test are indicated. Significant correlations are displayed in bold and with gray shading.

| **Effect on subjective**  **reports** | **Experiment 1: effect on estimated fingertip position** | | | **Experiment 2: effect on estimated fingertip position** | | | |
| --- | --- | --- | --- | --- | --- | --- | --- |
| *While listening to the*  *sound …* | *Ascend* | *Constant* | *Descend* | *Upw –Ascend* | *Upw - Descend* | *Downw - Ascend* | *Downw - Descend* |
| I felt pulling on my finger produced the sound | **rS=0.433** | rS=0.281 | rS=-0.363 | rS=0.123 | rS=0.162 | rS=-0.028 | rS=-0.126 |
|  | **p=0.044** | p=0.205 | p=0.097 | p=0.585 | p=0.470 | p=0.903 | p=0.575 |
| I felt my finger was longer | **rS=-0.473** | rS=-0.037 | rS=0.140 | rS=-0.371 | rS=0.136 | rS=-0.066 | rS=-0.064 |
|  | **p=0.026** | p=0.871 | p=0.533 | p=0.089 | p=0.545 | p=0.772 | p=0.776 |
| I felt my finger was  shorter | rS=0.149 | rS=-0.205 | **rS=0.495** | rS=0.200 | rS=-0.201 | rS=0.091 | rS=-0.099 |
|  | p=0.509 | p=0.361 | **p=0.019** | p=0.373 | p=0.369 | p=0.689 | p=0.660 |
| I felt my finger was rising | **rS=-0.500** | rS=0.079 | rS=0.253 | **rS=-0.502** | rS=0.105 | rS=-0.201 | rS=0.058 |
|  | **p=0.018** | p=0.725 | p=0.257 | **p=0.017** | p=0.642 | p=0.371 | p=0.798 |
| I felt my finger was  descending | **rS=-0.583** | rS=-0.070 | **rS=0.462** | rS=0.146 | rS=-0.281 | **rS=0.502** | rS=-0.049 |
|  | **p=0.004** | p=0.755 | **p=0.030** | p=0.517 | p=0.206 | **p=0.017** | p=0.830 |
| my finger felt stretched | rS=-0.138 | rS=0.158 | rS=-0.099 | rS=-0.290 | rS=0.198 | rS=0.073 | rS=-0.008 |
|  | p=0.539 | p=0.481 | p=0.660 | p=0.191 | p=0.377 | p=0.747 | p=0.973 |
| my finger felt squashed | rS=-0.146 | rS=-0.018 | rS=0.418 | rS=0.191 | rS=0.227 | rS=0.358 | rS=0.243 |
|  | p=0.518 | p=0.936 | p=0.052 | p=0.394 | p=0.311 | p=0.102 | p=0.276 |
| I couldn't tell how long  my finger was | rS=-0.421 | rS=-0.240 | **rS=0.430** | rS=-0.127 | **ol** | rS=0.204 | rS=-0.169 |
|  | p=0.051 | p=0.282 | **p=0.046** | p=0.574 | **p=0.010** | p=0.363 | p=0.453 |
| I couldn't locate the  position of my knuckle | rS=-0.054 | **rS=-0.471** | rS=0.280 | rS=0.269 | **rS=-0.509** | rS=-0.236 | rS=-0.033 |
|  | p=0.815 | **p=0.027** | p=0.207 | p=0.226 | **p=0.016** | p=0.290 | p=0.885 |
| I couldn't locate the  position of my fingertip | **rS=-0.475** | rS=-0.317 | **rS=0.565** | rS=0.131 | -0.390 | rS=-0.086 | rS=-0.155 |
|  | **p=0.026** | p=0.150 | **p=0.006** | p=0.561 | p=0.073 | p=0.704 | p=0.491 |
| the feeling from my finger  was unexpected | rS=-0.138 | rS=-0.267 | rS=0.382 | rS=-0.190 | **rS=-0.440** | rS=-0.050 | rS=0.308 |
|  | p=0.540 | p=0.229 | p=0.079 | p=0.398 | **p=0.040** | p=0.826 | p=0.163 |
| my finger felt like it  wasn't my own | rS=-0.204 | rS=-0.015 | **rS=0.648** | rS=0.234 | **rS=-0.424** | rS=0.062 | rS=0.228 |
|  | p=0.362 | p=0.947 | **p=0.001** | p=0.294 | **p=0.049** | p=0.784 | p=0.307 |
| my finger felt numb | rS=-0.279 | rS=-0.369 | **rS=0.506** | rS=-0.074 | rS=0.073 | rS=0.359 | rS=-0.294 |
|  | p=0.209 | p=0.091 | **p=0.016** | p=0.744 | p=0.746 | p=0.101 | p=0.184 |
| Effect on finger drawings | rS=-0.126 | rS=0.306 | rS=-0.298 | rS=-0.229 | rS=0.055 | rS=0.137 | rS=-0.281 |
|  | p=0.576 | p=0.166 | p=0.178 | p=0.304 | p=0.809 | p=0.555 | p=0.205 |

**Table S3. Results from Spearman correlations between estimates of knuckle position and subjective reports in Experiments 1 and Experiments 2.** For each correlation test, Spearman’s Rho (rS) and p-value of the test are indicated. Significant correlations are displayed in bold and with gray shading.

| **Effect on subjective reports** | **Experiment 1: effect on estimated knuckle position** | | | **Experiment 2: effect on estimated knuckle position** | | | |
| --- | --- | --- | --- | --- | --- | --- | --- |
| *While listening to the*  *sound …* | *Ascend* | *Constant* | *Descend* | *Upw –Ascend* | *Upw - Descend* | *Downw - Ascend* | *Downw - Descend* |
| I felt pulling on my finger produced the sound | rS=0.376 | rS=0.150 | **rS=-0.467** | rS=-0.074 | rS=0.090 | rS=0.058 | rS=0.063 |
|  | p=0.085 | p=0.504 | **p=0.029** | p=0.744 | p=0.692 | p=0.799 | p=0.780 |
| I felt my finger was longer | rS=-0.184 | rS=0.021 | rS=0.204 | rS=-0.276 | rS=-0.202 | rS=0.025 | **rS=0.425** |
|  | p=0.411 | p=0.927 | p=0.363 | p=0.213 | p=0.368 | p=0.912 | **p=0.049** |
| I felt my finger was shorter | rS=0.266 | rS=0.066 | rS=0.097 | rS=0.085 | rS=0.179 | rS=0.225 | rS=-0.026 |
|  | p=0.232 | p=0.771 | p=0.665 | p=0.707 | p=0.426 | p=0.313 | p=0.909 |
| I felt my finger was rising | rS=-0.210 | rS=0.106 | rS=0.200 | rS=-0.324 | rS=-0.163 | rS=0.035 | rS=0.252 |
|  | p=0.348 | p=0.639 | p=0.372 | p=0.141 | p=0.468 | p=0.877 | p=0.257 |
| I felt my finger was  descending | rS=-0.202 | rS=0.210 | rS=0.131 | rS=-0.125 | rS=-0.145 | rS=0.113 | rS=0.437 |
|  | p=0.367 | p=0.349 | p=0.562 | p=0.581 | p=0.519 | p=0.617 | p=0.042 |
| my finger felt stretched | rS=0.056 | rS=0.128 | rS=0.041 | rS=-0.171 | rS=-0.251 | rS=-0.110 | rS=0.301 |
|  | p=0.805 | p=0.570 | p=0.855 | p=0.446 | p=0.260 | p=0.627 | p=0.173 |
| my finger felt squashed | rS=0.244 | rS=0.098 | rS=0.075 | rS=-0.077 | rS=-0.107 | rS=0.071 | rS=0.101 |
|  | p=0.275 | p=0.664 | p=0.740 | p=0.732 | p=0.637 | p=0.754 | p=0.655 |
| I couldn't tell how long my  finger was | rS=-0.277 | rS=-0.211 | rS=0.205 | rS=-0.003 | rS=-0.103 | rS=-0.028 | rS=-0.044 |
|  | p=0.212 | p=0.346 | p=0.359 | p=0.990 | p=0.649 | p=0.902 | p=0.846 |
| I couldn't locate the position  of my knuckle | rS=0.152 | **rS=-0.451** | 0.192 | rS=0.051 | rS=-0.129 | rS=-0.259 | rS=-0.215 |
|  | p=0.510 | **p=0.035** | p=0.391 | p=0.820 | p=0.566 | p=0.244 | p=0.337 |
| I couldn't locate the position  of my fingertip | rS=-0.085 | rS=-0.229 | rS=0.314 | rS=0.219 | rS=-0.048 | **-0.429** | rS=-0.123 |
|  | p=0.707 | p=0.306 | p=0.154 | p=0.326 | p=0.831 | **p=0.046** | p=0.585 |
| the feeling from my finger  was unexpected | rS=-0.023 | rS=-0.275 | rS=0.014 | rS=0.251 | rS=0.148 | rS=-0.302 | rS=-0.151 |
|  | p=0.919 | p=0.216 | p=0.949 | p=0.260 | p=0.512 | p=0.172 | p=0.504 |
| my finger felt like it wasn't  my own | rS=0.266 | rS=-0.358 | rS=0.216 | rS=0.263 | rS=0.036 | rS=-0.254 | rS=0.034 |
|  | p=0.232 | p=0.102 | p=0.333 | p=0.236 | p=0.874 | p=0.255 | p=0.880 |
| my finger felt numb | rS=-0.161 | rS=-0.128 | rS=0.337 | rS=-0.219 | rS=0.097 | rS=0.307 | rS=0.121 |
|  | p=0.473 | p=0.571 | p=0.125 | p=0.327 | p=0.667 | p=0.165 | p=0.591 |
| Effect on finger drawings | rS=0.104 | rS=0.264 | rS=-0.035 | rS=-0.058 | rS=-0.237 | rS=0.156 | rS=0.330 |
|  | p=0.645 | p=0.235 | p=0.878 | p=0.798 | p=0.288 | p=0.499 | p=0.134 |

**Supplementary References**

1. Keenan, K. G., Collins, J. D., Massey, W. V., Walters, T. J., & Gruszka, H. D. Coherence between surface electromyograms is influenced by electrode placement in hand muscles*. Journal of neuroscience methods* **195**, 10-14 (2011).
2. Wiertlewski, M., Endo, S., Wing, A. M., & Hayward, V. Slip-induced vibration influences the grip reflex: A pilot study. In *World Haptics Conference (WHC)*, 2013 (pp. 627-632). IEEE. (2013).
3. Savitzky, A. & Golay, M. J. E. Smoothing and differentiation of data by simplified least squares procedures. *Analytical Chemistry* **36**, 1627-1639 (1964).
4. Olugbade, T. A., Aung, M. S., Bianchi-Berthouze, N., Marquardt, N., & Williams, A. C. Bi-modal detection of painful reaching for chronic pain rehabilitation systems, in *Proceedings of the 16th International Conference on Multimodal Interaction* 455-458 (ACM, 2014).
